# Simultaneous Quantification of Six Bioactive Components in Decoction of Ziziphi spinosae Semen Using Ultrahigh Performance Liquid Chromatography Coupled with Triple-Quadrupole Mass Spectrometry

**DOI:** 10.1155/2018/8397818

**Published:** 2018-11-01

**Authors:** Xiao Liu, Xiaochai Zhu, Hui Zhu, Li Xie, Jia Ma, Yinghui Xu, Qigang Zhou, Zejun Wu, Baochang Cai

**Affiliations:** ^1^School of Pharmacy, Nanjing University of Chinese Medicine, Nanjing, China; ^2^Fushun Central Hospital, Fushun, China; ^3^Department of Pharmacology, Pharmacy College, Nanjing Medical University, Nanjing, China

## Abstract

This paper was conducted to develop a method containing ultrahigh performance liquid chromatography coupled with triple-quadrupole tandem mass spectrometry for simultaneous quantification of six bioactive components in the decoction of Ziziphi spinosae Semen. Analysis was performed on an Agilent ZORBAX Extend-C_18_ column (2.1 × 100 mm, 1.8 *μ*m) and eluted with a mobile phase system consisting of acetonitrile and water under a gradient program with a flow rate of 0.3 ml/min. The injection volume was 2 *μ*l. Multiple-reaction monitoring scanning detection was employed for quantification with an electrospray ion source in the negative ion mode. All the six compounds showed good linearities (*r* ≥ 0.9996). The LODs of the six bioactive compounds were 0.039 ng/ml, 0.092 ng/ml, 3.112 ng/ml, 2.131 ng/ml, 0.099 ng/ml, and 0.071 ng/ml for spinosin, 6‴-feruloylspinosin, jujuboside A, jujuboside B, camelliaside B, and betulinic acid, respectively. The LOQs were 0.118 ng/ml, 0.276 ng/ml, 9.336 ng/ml, 6.393 ng/ml, 0.299 ng/ml, and 0.213 ng/ml for spinosin, 6‴-feruloylspinosin, jujuboside A, jujuboside B, camelliaside B, and betulinic acid, respectively. According to our knowledge, it was the first time to establish a method with high efficiency and accuracy for the quantification of six bioactive components in the decoction of Ziziphi spinosae Semen, which would provide references for quality control and evaluation of Ziziphi spinosae Semen.

## 1. Introduction

Ziziphi spinosae Semen (ZSS; suanzaoren in Chinese), the seeds of *Ziziphus jujuba* Mill. var. spinosa (Bunge) Hu ex H. F. Chou, has been used as a typical sedative-hypnotic traditional Chinese medicine (TCM) in clinic for thousands of years in China [1, 2]. There are many different categories of chemical constituents in ZSS, including flavonoids, saponins, and triterpenoids. Spinosin, jujuboside A, and jujuboside B were reported to be active components which exerted sedative-hypnotic effect [3–8]. It was found that the sedative-hypnotic effect of ZSS might be due to the action of its chemical constitutions, such as spinosin, jujuboside A, and jujuboside B, on serotonergic system and GABA receptors [9–11]. Several studies have reported the analysis of relative chemical constituents in ZSS; however, the limitation of the performance coming from traditional detectors, such as UV and ELSD, made it quite difficult to detect a variety of complex components in ZSS quickly and accurately [12, 13]. Recently, tandem mass spectrometry with high sensitivity and selectivity has become the mainstream detection method due to its advantages in simultaneous quantitative and qualitative analyses. The combination of tandem mass spectrometry with UHPLC made it possible for the effective and reliable detection of complex constitutions in TCM, which would compensate for the 3 shortcomings in the previous research studies [14–17]. In fact, chemical constituents of ZSS have been studied in some previous research studies [18, 19], but none of them could determine the contents of its active components efficiently due to the diversity of these constituents.

Decoction was the most common and traditional using form during TCM clinical application [20]. However, organic solvents were used for sample extraction in most of the existing research studies [21], which was different from TCM actual application in clinics. And this might lead to a limitation of these research studies for guiding ZSS clinical application and quality control.

## 2. Experimental

### 2.1. Materials and Reagents

Methanol (LC-MS grade) and acetonitrile (LC-MS grade) were obtained from E. Merck (Merck, Darmstadt, Germany). Ultrapurified water (18.25 Ω) was daily prepared with a Milli-Q water purification system (Millipore Corporation, Bedford, MA, USA). All the other regents were of analytical purity and commercially available.

Reference standards including spinosin (batch no. S-043-150626), 6‴-feruloylspinosin (batch no. A-011-170731), jujuboside A (batch no. S-045-160809), and jujuboside B (batch no. S-046-151205) were purchased from Chengdu Ruifensi Biological Technology Co. Ltd. Camelliaside B (batch no. 170313-080) and betulinic acid (batch no. JYB 201701) were purchased from JinYiBai Biological Technology Co. Ltd. The purity of these reference standards was all above 98.0%.

Ten different batches of ZSS samples collected from Shandong (batch no. 1-5), Shanxi (batch no. 6-7), and Hebei (batch no. 8-10) provinces, which are the main production regions of ZSS in China, were used as experimental materials after a careful quality evaluation according to the Chinese Pharmacopoeia 2015 edition-part one [1].

### 2.2. Preparation of Standard Solutions

The stock resolutions of 6 components were independently prepared. Then, the mixed reference standard solution was obtained by moderate dilution of the stock solution with methanol. The concentrations of the 6 components in the final mixed reference standards solution used for identification and quantification were 6.312, 5.885, 0.991, 0.668, 0.640, and 4.504 *μ*g/ml. A series of reference standard solutions with six different concentrations were prepared from the final mixed reference standard solution by diluting 1, 2, 4, 8, 16, and 32 times, respectively. Finally, the concentration ranges of each reference standard in the series of reference standard solutions were 0.197–6.312 *μ*g/ml, 0.184–5.885 *μ*g/ml, 0.031–0.991 *μ*g/ml, 0.021–0.668 *μ*g/ml, 0.020–0.640 *μ*g/ml, and 0.141–4.504 *μ*g/ml, respectively.

### 2.3. Sample Preparation

According to the experience of clinical application of ZSS, 10 g dried powder of ZSS (60 mesh sieve passed) was accurately weighed and decocted twice with boiling water (1 : 10, *w/v* and 1 : 8, *w/v*, resp.), each for 1 h. Solutions were filtered with 4-layer meshes and merged together. Water was added to the merged decoction to fix a final volume of 200 ml to obtain the decoction of ZSS. 0.2 ml of the decoction sample was transferred into a 5 ml volumetric flask, and then appropriate methanol was added to the fixed volume and mixed. Appropriate amount of the mixture was removed and centrifuged at 12000 r/min for 5 min. The supernatant liquor was taken out, and 2 *μ*l of which was finally injected into the UHPLC-QQQ-MS/MS system for analysis.

### 2.4. UHPLC-QQQ-MS/MS Conditions

A Shimazu 30A UHPLC (Shimadzu, Japan) equipped with a binary solvent system was used to perform chromatographic separation. An Agilent C_18_ column (2.1 mm × 100 mm, 1.8 *µ*m) was used at 25°C. A linear gradient program was performed with the mobile phases consisting of eluent A (water) and eluent B (acetonitrile) as follows: 0–1.2 min, 5–31% B; 1.2–2.5 min, 31–95% B; 2.5–4.5 min, 95–100% B; and 4.5–6 min, 100–100% B. The flow rate was 0.3 ml/min.

An MS/MS system (Triple Quad 5500, AB Sciex, USA) with an electrospray ionization source (ESI) was used to perform analysis of the six bioactive components in decoction of ZSS. The detection was performed under negative ion mode with an MRM detection way. The acquisition parameters of mass spectrometry are listed as follows: atomization gas (N_2_) of 55 psi; auxiliary heating gas of 55 psi; curtain gas of 35 psi; turbospray temperature of 550°C; and ion spray voltage floating of −4500 V. The MRM transitions were tuned to the optimal for meeting the standard of the experiment. Also, declustering potential and collision energy parameters of the six components were debugged to optimal in order to meet the purpose of accurate detection of all the components.

### 2.5. Quantification Analysis

AB Sciex MultiQuant 2.1 Software was applied to perform the quantification of the six components. Peak area ratios were calculated and plotted against concentrations by using the software.

## 3. Results and Discussion

### 3.1. Optimization of the Chromatographic and Spectrometric Conditions

**Figure 1:**
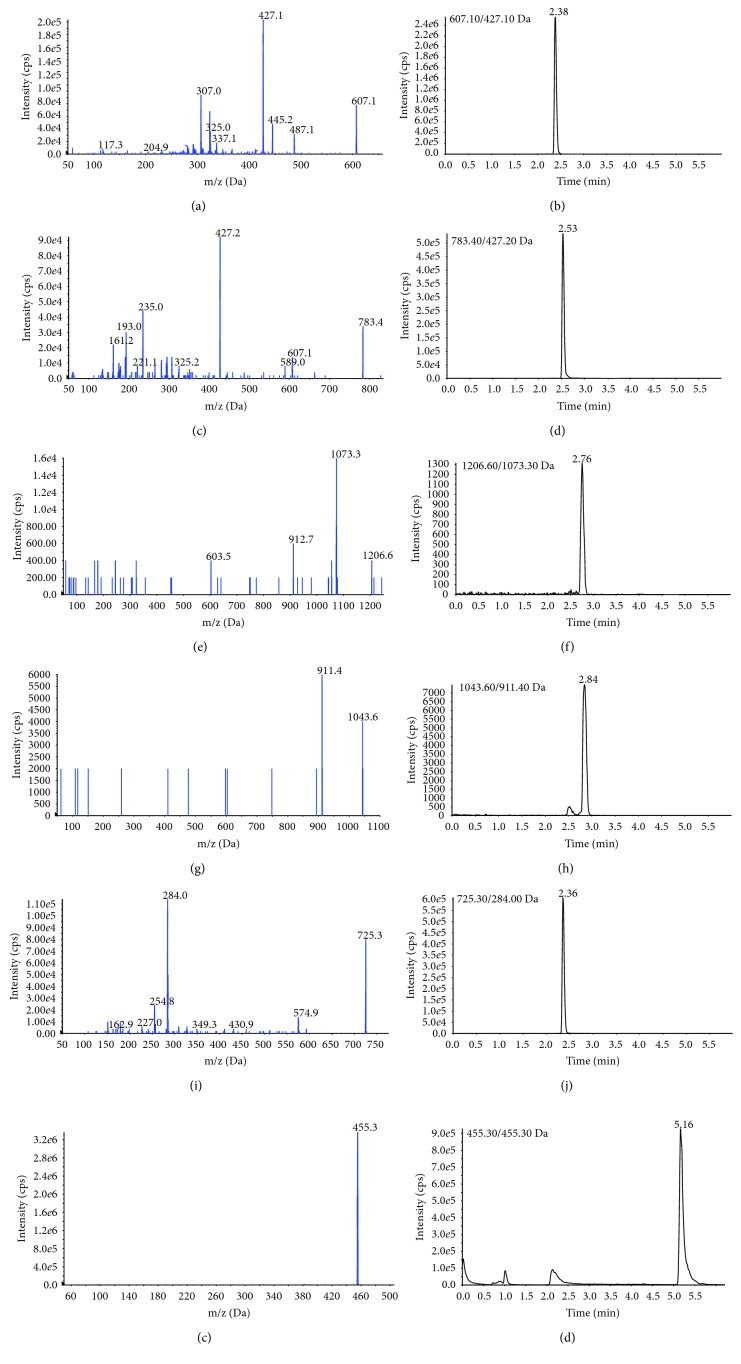
The representative MS^2^ spectrum of (a) spinosin; (c) 6‴- feruloylspinosin; (e) jujuboside A; (g) jujuboside B; (i) camelliaside B; and (k) betulinic acid, and MRM chromatograms of (b) spinosin; (d) 6‴-feruloylspinosin; (f) jujuboside A; (h) jujuboside B; (i) camelliaside B; and (l) betulinic acid of six analytes in reference standards under experimental conditions.

It was reported in some previous studies that the six components analyzed in this study were active components which exerted sedative-hypnotic effect [3–8]. Using the UHPLC-QQQ-MS/MS method developed in this experiment, the six components with different types of structures could be well separated and quantified within 6 minutes. Methanol or acetonitrile as the organic phases and water or 0.1% formic acid in water (v : v) as the aqueous phases were tested to obtain good chromatographic peaks. In order to accurately determine the six components, the ion fragments of MRM transition of these analytes were detected. All the chemical information of these components was further confirmed by comparisons of retention time to the mixed reference standard. The detailed UHPLC-ESI-MS/MS detection parameters of the six components are shown in Table 1. The representative MS^2^ spectrum and MRM chromatograms of six analytes in this experiment are shown in Figure 1.

### 3.2. Assay Validation

#### 3.2.1. Linearity and Range

AB Sciex MultiQuant 2.1 software was used to determine regression lines as well as to ensure precision. The mixed reference standard solution was diluted to a series of standard solutions with different concentrations in accordance with gradients of 1, 2, 4, 8, 16, and 32 times, which could be used to make the standard curves. At the same time, the reference standard solution was gradually diluted and detected, and the limitation of quantitation (LOQ) and limitation of detection (LOD) were performed. LOQ was determined as the concentration whose *S*/*N* was 10, and LOD was determined as the concentration whose *S*/*N* was 3. All the *r* values were above 0.999 0 in this experiment, which indicated that the linear relationship of this method for each analyte was good. The regression equations and linear ranges of all the detected components are shown in Table 2.

#### 3.2.2. Precision Test

Proper amount of mixed reference standard solution was taken and detected for six times under the testing conditions. The peak areas of the six components were recorded, which could be used to investigate the precision by calculating the RSDs. The results showed that the RSDs of the six components were 0.60%, 2.51%, 2.75%, 2.91%, 1.12%, and 2.62%. The precision of this method was excellent.

#### 3.2.3. Repeatability Test

Six copies of ZSS powder, taken from the same batch, each for 10 g, were used to prepare the trial solution in accordance with the method in Section 2.3. Peak areas of the six components were recorded to investigate the repeatability of the method by calculating RSDs. The results showed that RSDs were 1.20%, 2.64%, 2.60%, 2.21%, 2.98%, and 1.18%, indicating that the repeatability of this method met the requirement.

#### 3.2.4. Stability Test

The trial solution was taken from the samples prepared in Section 2.3 randomly and tested at 0, 2, 4, 8, 12, 24, and 48 h under the analytical conditions. The peak areas of the six components were recorded to calculate the RSDs, which were used to investigate the stability of the samples. The result that the RSDs of the six components were 2.96%, 2.53%, 2.82%, 2.10%, 2.31%, and 2.72% indicated that the stability of the samples was good.

#### 3.2.5. Recovery Test

Dried powder of ZSS (5 g, 60 mesh sieve passed) was accurately weighed, and fair level of reference standard solution was added, which would be then prepared as the trial solution according to Section 2.3. Determination was performed under the analytical conditions, and the peak areas of the six components were recorded in order to calculate RSDs and recoveries. The data shown in Table 3 indicated that the RSDs and recoveries for six analytes were eligible.

#### 3.2.6. Quantification of the Six Components in ZSS Samples

Ten different batches of ZSS samples collected from their main producing regions in China were prepared as the trial solution in accordance with the method in Section 2.3. Determination of the components was performed under the analytical conditions given in Section 2.4, and the peak areas which would be used to calculate the mass fraction were recorded. The contents of the six bioactive constituents of the decoctions of ZSS samples from three different producing regions were in the ranges of 1375.17–2612.84, 297.27–788.05, 60.20–112.75, 47.34–114.60, 10.17–46.13, and 141.78–616.83 *μ*g/g (Table 4). Interestingly, among all these six components determined, the contents of spinosin and jujuboside A, the most important active constituents which exerted sedative-hypnotic effects, were relatively stable in the ten batches of ZSS samples from three different regions. As for the other four components, their contents fluctuated more clearly in these samples as the regions changed. Taking a comprehensive evaluation into consideration, the contents of the six bioactive ingredients in the samples from Hebei province (samples with the No. from 8 to 10) were the highest, followed by the samples from Shandong province (samples with the No. from 1 to 5) being the second one. The results of this experiment indicated that the contents of bioactive components were quite different in ZSS from different regions. Therefore, the origin might be a key factor that should be considered when ZSS was clinically applied for its sedative-hypnotic action.

## 4. Conclusions

A rapid and accurate method for determination of six bioactive components in the decoction of ZSS was established for the first time in this paper. Besides accurate and quick quantitation, the mass information and ion fragments of MRM transition could provide meaningful reference for the identification of the analytes. It was hoped that the analytical method developed would provide a reference for the quality control and evaluation of ZSS. At the same time, the results that great differences existed in the contents of the six bioactive components in the samples from different regions indicated that the origin factor might be related to ZSS clinical efficacy.

## Figures and Tables

**Table 1:** HPLC-ESI-MS/MS detection parameters of the six components.

Analyte	Mass data	*t*_R_ (min)	Ion mode	MS^1^ (*m/z*)	MS^2^ (*m/z*)	DP/V	CE/eV
Spinosin	608.1741	2.40	ESI^−^	607.10	427.10	−26.46	−41.60
6‴-Feruloylspinosin	784.2215	2.54	ESI^−^	783.40	427.20	−25.83	−50.92
Jujuboside A	1207.3533	2.75	ESI^−^	1206.60	1073.30	−60.00	−60.00
Jujuboside B	1044.5505	2.83	ESI^−^	1043.60	911.40	−27.20	−49.77
Camelliaside B	726.2007	2.36	ESI^−^	725.30	284.00	−23.72	−53.07
Betulinic acid	456.3604	5.16	ESI^−^	455.30	455.30	−97.00	−20.00

**Table 2:** Calibration curves, correlation coefficients (*r*), and linear ranges of the six components.

Analyte	Calibration curve	*r*	Linear range (*μ*g/ml)	LODs (ng/ml)	LOQs (ng/ml)
Spinosin	*y* = 2.08*e*^6^*x* + 7.35*e*^5^	0.999 6	0.197–6.312	0.039	0.118
6‴-Feruloylspinosin	*y* = 1.32*e*^6^*x* + 3.79*e*^5^	0.999 7	0.184–5.885	0.092	0.276
Jujuboside A	*y* = 1.05*e*^4^*x* − 241.13	0.999 6	0.031–0.991	3.112	9.336
Jujuboside B	*y* = 7.28*e*^4^*x* − 2926.02	0.999 6	0.021–0.668	2.131	6.393
Camelliaside B	*y* = 1.10*e*^7^*x* + 6.01*e*^5^	0.999 7	0.020–0.640	0.099	0.299
Betulinic acid	*y* = 1.00*e*^7^*x* + 9.11*e*^5^	0.999 8	0.141–4.504	0.071	0.213

**Table 3:** Recovery data of the six analytes (*n*=6).

Analyte	Sample content (*μ*g)	Actual amount of control (*μ*g)	Amount of control found (*μ*g)	Recovery (%)	RSD (%)
Spinosin	15.25	15.58 ± 0.0.26	15.60 ± 0.19	100.14 ± 1.00	0.97
6‴-Feruloylspinosin	7.50	7.34 ± 0.25	7.33 ± 0.24	99.88 ± 0.02	1.81
Jujuboside A	1.32	1.33 ± 0.19	1.35 ± 0.16	98.21 ± 0.03	2.64
Jujuboside B	0.88	0.84 ± 0.06	0.84 ± 0.05	99.90 ± 0.03	2.88
Camelliaside B	0.79	0.81 ± 0.02	0.81 ± 0.03	99.81 ± 0.03	2.97
Betulinic acid	2.90	2.86 ± 0.05	2.88 ± 0.05	100.70 ± 0.01	1.31

**Table 4:** The contents of the 6 components in the decoction of ZSS samples (*μ*g/g).

No.	Spinosin	6‴-Feruloylspinosin	Jujuboside A	Jujuboside B	Camelliaside B	Betulinic acid
1	2069.00	783.50	105.75	80.40	58.92	403.40
2	1774.83	576.17	83.05	67.97	30.41	598.50
3	2063.67	622.50	78.52	80.62	37.89	141.78
4	1375.17	297.27	60.20	47.77	10.17	440.30
5	1507.17	300.08	57.80	61.18	13.19	616.83
6	1748.33	436.28	66.70	50.51	25.60	469.35
7	1520.83	304.38	67.37	80.17	12.54	177.92
8	2123.97	598.51	88.95	47.34	31.88	293.07
9	2209.67	639.33	95.22	90.38	36.33	228.03
10	2612.84	788.05	112.75	114.60	46.13	187.72
Average	1900.55	534.61	81.63	72.09	30.31	355.69
SD	363.56	180.19	18.04	20.38	14.84	165.92

## Data Availability

The data used to support the findings of this study are included within the article.

## Abbreviations

UHPLC-QQQ-MS: Ultrahigh performance liquid chromatography method coupled with triple-quadrupole mass spectrometry
ZSS: Ziziphi spinosae Semen
TCM: Traditional Chinese medicine
UV: Ultraviolet
ELSD: Evaporative light-scattering detector
MRM: Multiple-reaction monitoring.
